# Feasibility of isometric spinal muscle training in patients with bone metastases under radiation therapy - first results of a randomized pilot trial

**DOI:** 10.1186/1471-2407-14-67

**Published:** 2014-02-05

**Authors:** Harald Rief, Georg Omlor, Michael Akbar, Thomas Welzel, Thomas Bruckner, Stefan Rieken, Matthias F Haefner, Ingmar Schlampp, Alexandros Gioules, Daniel Habermehl, Friedbert von Nettelbladt, Jürgen Debus

**Affiliations:** 1 Department of Radiation Oncology, University Hospital of Heidelberg, Im Neuenheimer Feld 400, 69120 Heidelberg, Germany; 2 Department of Medical Biometry, University Hospital of Heidelberg, Im Neuenheimer Feld 305, 69120 Heidelberg, Germany; 3 Department of Orthopaedics and Trauma Surgery, University Hospital of Heidelberg, Schlierbacherstrasse 120a, 69118 Heidelberg, Germany

**Keywords:** Bone metastases, Spine, Physical exercise, Stability, Isometric training

## Abstract

**Background:**

Spinal bone metastases are commonly diagnosed in cancer patients. The consequences are pain both at rest and under exercise, impairment of activities of daily life (ADL), reduced clinical performance, the risk of pathological fractures, and neurological deficits. The aim of this randomized, controlled pilot trial was to investigate the feasibility of muscle-training exercises in patients with spinal bone metastases under radiotherapy. Secondary endpoints were local control, pain response and survival.

**Methods:**

This study was a prospective, randomized, monocentre, controlled explorative intervention trial to determine the multidimensional effects of exercises for strengthening the paravertebral muscles. On the days of radiation treatment, patients in the control group were physically treated in form of respiratory therapy. Sixty patients were randomized between September 2011 and March 2013 into one of the two groups: differentiated resistance training or physical measure with thirty patients in each group.

**Results:**

The resistance training of the paravertebral muscles was feasible in 83.3% of patients (n = 25). Five patients died during the first three months. The exercise group experienced no measurable side effects. “Chair stand test” in the intervention group was significant enhanced with additionally improved analgesic efficiency. Patients in intervention group improved in pain score (VAS, 0–10) over the course (p < .001), and was significant better between groups (p = .003) after 3 months. The overall pain response showed no significant difference between groups (p = .158) There was no significant difference in overall and bone survival (survival from first diagnosed bone metastases to death).

**Conclusions:**

Our trial demonstrated safety and feasibility of an isometric resistance training in patients with spinal bone metastases. The results offer a rationale for future large controlled investigations to confirm these findings.

**Trial registration:**

Clinical trial identifier NCT01409720.

## Background

The vertebral column is the main localization of bone metastases, where they frequently indicate an advanced stage of a malignant primary disease [1,2]. Two thirds of all tumor patients are estimated to develop bone metastases in the course of their disease [3]. The clinical symptoms include pain at rest and under exercise, but also impaired activity of daily life (ADL), the risk of pathological fractures, and neurological deficits. Standard clinical care often includes patient immobilization either by means of an orthopedic thoracic corset or by confining the patient to bed in order to prevent pathological fractures. Regarding pain therapy and recalcification of former osteolytic lesions, palliative radiotherapy (RT) represents an effective treatment option [4].

As the central axial organ of the human body, the vertebral column is involved in all physical movements and any spinal impairment with critically limited patient mobility. The paravertebral muscles greatly contribute to relief of pressure on the spine and, therefore, have a share in the realization of mobility. For this reason, exercise-related interventions have until now been excluded in patients with bone metastases, and the literature does not describe any targeted training-therapeutic measures involving isometric muscle exercise in these patients. There are, however, numerous findings that indicate the positive effect of targeted physical training measures in tumor patients regarding practicability, pain, and mobility [5-10]. Correspondingly, the effect of resistance training as an adjunct to RT in patients with bone metastases is still unknown. In these patients with a generally advanced stage of the tumor, a painful vertebral column, and in a reduced general physical condition, this prospective trial presents a challenge in the investigation of the feasibility of a targeted, routine, and differentiated training program for strengthening the paravertebral muscles in patients with bone metastases of the vertebral column. An aspect of critical importance is first to distinguish between stable and unstable lesions, since an acute instability represents a contraindication for resistance training in patients with bone metastases. The aim of our trial was to analyze the feasibility of a combination therapy, in patients with spinal metastases in order to promote early mobility.

## Methods

### Subjects, recruitment strategy, and eligibility for enrolment

**Figure 1:**
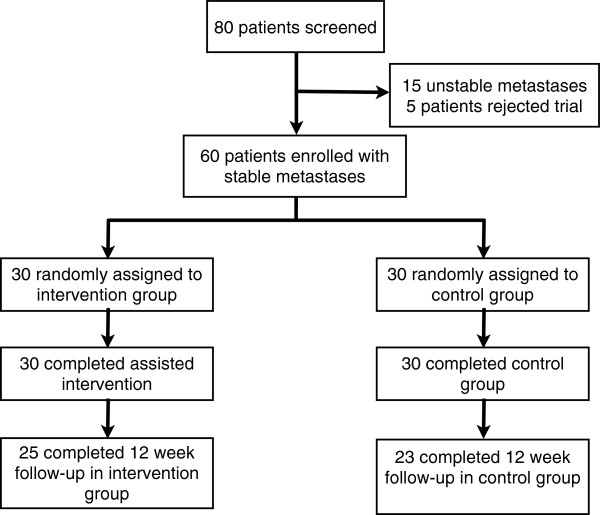
Flow of participants through the trial.

From September 2011 to March 2013, 80 patients with a histologically confirmed tumor diagnosis and also solitary or multiple bone metastases of the thoracic or lumbar segments of the vertebral column or of the sacral region were screened in our department. Initially all patients were diagnosed with painful bone metastases requiring RT. Inclusion criteria were an age of 18 to 80 years, a Karnofsky performance score [11] ≥ 70, written declaration of informed consent, and already initiated bisphosphonate therapy. Furthermore, only patients with stable vertebral-body lesions were included. This was diagnosed independently by a specialist for radiology as well as by a specialist for orthopedic surgery. Only a metastasis classified by both specialists as “stable” was suggested eligible for inclusion. Patients with significant neurological or psychiatric disorders – including dementia and epilepsy, contractual incapacity, and diagnosed vertebral-body instability or involvement of the cervical spine were excluded. Fifteen patients were excluded due to unstable metastases, and five patients declined to participate in the study. Sixty patients fulfilled the inclusion and exclusion criteria and were enrolled into the trial (Figure 1). The study was approved by the Heidelberg Ethics Committee (Nr. S-316/2011).

### Design, randomized allocation, and procedures

**Figure 2:**
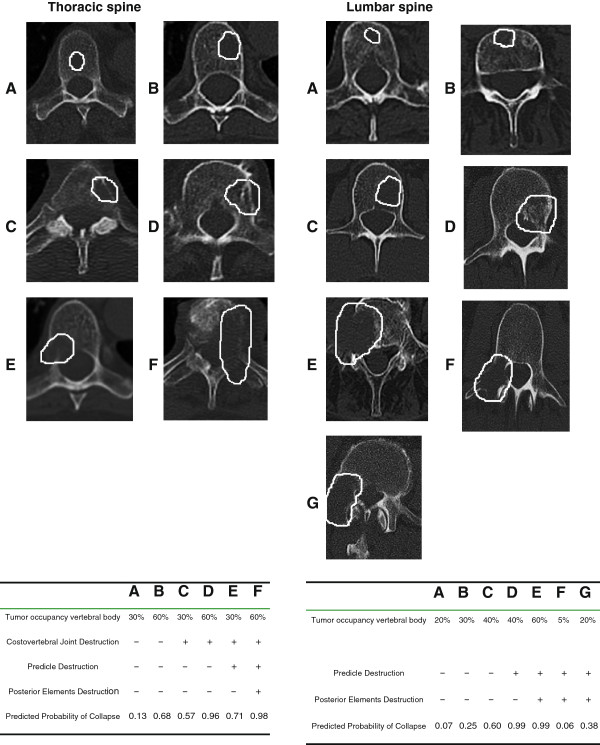
**Taneichi score **[20]**.**

This was a randomized, monocentre, controlled, explorative intervention study with the intention to determine the feasibility of a resistance training program for strengthening the paravertebral muscles in patients with spinal bone metastases under RT. The intervention was conducted initially under guidance and was subsequently continued by the individuals themselves. The control group underwent physical therapy in the form of respiration exercises and “hot roll” treatments. The patients were subjected to a staging of their vertebral column within the context of the computer tomography scans (CT) designed to plan the RT schedule prior to enrolment into the trial. In this examination the osteolytic metastases in the thoracic and lumbar spine were classified according to Taneichi [12] and correspondingly classified as “stable” or “unstable”. The subtypes A-C were defined as “stable” in thoracic and lumbar spine (Figure 2). Osteoblastic and mixed metastases were assessed separately, since the Taneichi score can only be used for the assessment of osteolytic metastases. After completion of the measurement of the baseline findings, patients with stable bone metastases were allocated to one of the two treatment groups by randomization. A block randomization approach with block size 6 was used to ensure that the two intervention groups were balanced equally. A random list was used SAS 9.1. After the baseline measurements, the patients were assigned to the respective treatment arms on a 1:1 basis according to the randomization list. The randomization procedure was carried out by a central office. The data of the patient records were collected by the authors. The evaluation included all recorded data up to the time of the three-month follow-up interval. The data of the patient characteristics are presented in summary Table 1.

**Table 1:** Patient characteristics at baseline

	**Intervention group (n = 30)**		**Control group (n = 30)**		**p-value**
	**n**	**%**	**n**	**%**	
Age (years)					
Mean (SD)	61.3 (10.1)		64.1 (10.9)		0.304
Gender					
Male	14	46.7	19	63.3	0.195
Female	16	53.3	11	36.7	
Weight (kg, SD)	74.3 (14.7)		72.7 (12.1)		0.553
Height (cm, SD)	171.2 (8.3)		172.3 (7.5)		0.906
Body mass index					
Mean (SD)	25.3 (4.5)		24.4 (3.6)		0.559
Karnofsky-index (median, range)	80 (70–100)		80 (70–100)		1.000
Primary site					
Lung cancer	12	9.2	8	26.6	0.320
Breast cancer	5	16.7	6	20.1	0.542
Prostate cancer	5	16.7	9	30.1	0.156
Melanoma	1	3.3	1	3.3	1.000
Renal cancer	1	3.3	2	6.7	0.875
Other	6	20.1	4	13.4	0.325
Localization metastases					0.717
Thoracic	17	56.7	14	46.7	
Lumbar	9	30.0	13	43.3	
Thoracic and lumbar	2	6.7	2	6.7	
Sacrum	2	6.7	1	3.3	
Number metastases					0.257
Mean (range)	1.4 (2–4)		1.7 (1–5)		
Solitary	22	73.3	18	60.0	
Multiple	8	26.7	12	40.0	
Type of metastases					0.961
Mixed	2	6.7	2	6.7	1.000
Osteoblast	9	30.0	10	33.3	0.956
Osteolytic	19	63.3	18	60.0	.0932
Distant metastases at baseline					
Visceral	12	40.0	5	16.7	0.045
Brain	3	10.0	3	10.0	1.000
Lung	7	23.3	4	13.3	0.320
Tissue	8	26.7	6	20.0	0.542
Hormonotherapy	10	33.3	16	53.3	0.118
Immunotherapy	7	23.3	5	16.7	0.519
Chemotherapy	25	83.3	20	66.7	0.136
Pathological fracture at baseline	6	20.0	9	30.0	0.379
Neurological deficit	0	0.0	2	6.7	0.150
Orthopedic corset at baseline	7	23.3	5	16.7	0.519
Radiotherapy dose completed (Gy)					0.136
Single dose (median, range)	3 (2–4)		3 (2–4)		1.000
Cumulative dose (median, range)	30 (30–40)		30 (30–40)		1.000

*Abbreviations*: *SD* Standard deviation.

### Study interventions

Arm A (intervention group, differentiated resistance training) and in Arm B (control group, physical “respiratory” measure) each consisted of 30 patients. The interventions started on the same day with RT and were performed on each of the treatment days (Monday until Friday) over a two-week period, independent of the number of RT fractions. The sports intervention lasted approximately 30 min, the “respiratory” measure approximately 15 min [13]. The patients exhibit differences in terms of age, physical constitution, gender, stage of tumor, general state of health, bone density, and pain symptoms, which is why the muscle-exercise concept was kept as simple as possible. Since the site of the bone metastases differed from patient to patient, three different exercises were enacted to ensure an even isometric training of the muscles along the entire vertebral column. The participants of the control group were given physical therapy in the form of respiration exercises and “hot roll” treatments also for a period of two weeks. A detailed report of the sports intervention and its application has already been published [14].

After completion of RT schedule or, respectively, after two weeks, patients in the training group were guided to continue exercises, which were demonstrated to them by their therapist in the one-on-one situation, on their own at home for a further twelve weeks. The training exercises were documented. The patients in the control group did not carry out any further measures at home after the two-week therapy period. The target parameters were measured at the start of RT (t_0_), at the end (t_1_), and after twelve weeks (t_2_). The target parameters comprise the documentation of the training program, the pain score according to the visual analog scale (VAS), the completion of the activity questionnaire, and the recording of patient-specific data. Since it was not possible to quantitatively measure the power of the paravertebral muscles as a baseline value and to monitor the success of the training, the so-called “chair stand” test [15] was carried out at all measurement intervals. In this test the subjects were asked to stand up from the sitting position as many times as possible within a period of 30 seconds, with the number of times being recorded as the score.

### Assessment of the primary and secondary endpoints

The aim of the trial was to evaluate the feasibility of the defined training program. The feasibility as the primary endpoint was defined as the completion of the training program up to three months after the end of RT (t_2_). In addition, the evaluation of the mobility aspect included the performance of the chair-stand test at the individual investigation intervals. The item taken as the secondary endpoint was the activity of the patients as documented on an activity questionnaire specially designed for this trial and completed on the individual days of examination (Table 2). We created questions independently which were relevant for these palliative patients. Furthermore, the local control was assessed by means of CT images taken prior to and three months after RT. The pain response was documented on the VAS (range 0–10). Complete response (CR) was defined as VAS = 0 after three months, partial response (PR) as an improvement by at least two score points after three months, according to the international consensus response categories by Chow et al. [16]. Overall survival (OS) was defined as time from initial diagnosis until death, bone survival as time from initial diagnosis of spinal bone metastasis until death.

**Table 2:** Questions of the activity questionnaire

	
1.	I don’t have any trouble putting on my socks/shoes on my own. (absolutely true = 1 to absolutely false = 6)
2.	I don’t have any trouble putting on a t-shirt on my own. (absolutely true = 1 to absolutely false = 6)
3.	I have trouble getting up from a low chair. (absolutely true = 1 to absolutely false = 6)
4.	I don’t have any trouble getting into a car. (absolutely true = 1 to absolutely false = 6)
5.	The longest distance I can currently walk is approx. <100 m; 100–500 m; 500-1000 m; 1–2 km; 2–5 km; > 5 km
6.	After covering this distance I’m thoroughly exhausted. (absolutely true = 1 to absolutely false = 6)
7.	I’m still capable of riding a bicycle yes = 1; no = 0
8.	The longest riding distance I’m still capable of is approx. <2 km; 2–5 km; 5–10 km; 10–15 km; 15–20 km; >20 km
9.	After riding this distance I’m thoroughly exhausted. (absolutely true = 1 to absolutely false = 6)
10.	I can walk up stairs from one storey to the next with ease. (absolutely true = 1 to absolutely false = 6)
11.	I don’t have any trouble carrying a shopping basket approx. 50 m. (absolutely true = 1 to absolutely false = 6)

### Compliance with the intervention

During the two-week period of RT, patients in the training group (Arm A) performed exercises under the guidance of a physiotherapist. The patients were then requested to carry out the defined training program in their home setting three times a week and to document the exercises themselves. We could improve the compliance with no implements for home training, and the exercises were practicable easily. This training schedule was verified at the t_2_ follow-up interval.

### Radiotherapy

Radiotherapy was performed in our department. After virtual simulation was performed to plan the radiation schedule, radiotherapy was carried out over a dorsal photon field of the 6MV energy range. Primary target volume (PTV) covered the specific vertebral body affected as well as the ones immediately above and below. In Arm A, 24 patients (80%) were treated with 10 × 3 Gy, three patients (10%) with 14 × 2.5 Gy, and three patients (10%) with 20 × 2 Gy. In Arm B, the RT protocols for 28 patients (93.4%) were 10 × 3 Gy, for one patient (3.3%) 14 × 2.5 Gy, and for one patient (3.3%) 20 × 2 Gy. The median single dose was 3 Gy (range 2–3 Gy), the median total dose 30 Gy (range 20–35 Gy). The single and total doses were decided separately for each patient, depending on the histology, the patient’s general state of health, and on the current staging and the corresponding prognosis.

### Sample calculation and statistical analysis

The total number of patients undergoing radiotherapy in the radiation oncology department of the Heidelberg University Clinic for metastatic processes in the vertebral column in the recruitment period is approximately 120, about 90 of whom shall fulfill the inclusion criteria. On account of the explorative character of this study it was not possible to estimate the total number of cases; with a scheduled number of 30 patients per group, it will, however, be possible to detect a standardized mean-value effect of 0.8 with a power of 80% and an α significance level of 5%. All variables were analyzed descriptively by tabulation of the measures of the empirical distributions. According to the scale level of the variables, means, standard deviations, medians as well as minimum and maximum or absolute and relative frequencies, respectively, will be reported. The results are reported as p-values. For all analysis, a p-value of 0.05 or less was considered significant. All statistical analyses were done using SAS software Version 9.1 (SAS Institute, Cary, NC, USA).

## Results

Groups were balanced at baseline. The median follow-up was 3.3 months for both groups (range 2.8-4.0 months). Patients in the intervention group (Arm A) completed the isometric resistance training of the autochthonous muscles in 83.3% (n = 25) of all cases. Five patients (16.7%) died within the first twelve weeks following RT due to tumor progression. In Arm B, 7 patients (23.3%) died within 3 months. In the intervention group, fatigue and psychological stress decreased during the training program (p < .001), and there was a significant difference between baseline and after 3 month in both parameters (p < .001) (Table 3). Pathological fractures or progression of a neurological deficit did not occur in both groups. Patients in Arm A improved significantly in the chair-stand test (p < .0001) over the course, and between groups in favor to intervention group (p < .001). No significant difference could be measured in the control group (p = .525) (Table 4). This result is also reflected in the evaluation of the activity questionnaire (Tables 2 and 5). After three months, none of the patients (n = 0; 0%) in the intervention group required an orthopedic thoracic corset any longer, while the difference in Arm B was unchanged (n = 4; 17.4%).

**Table 3:** Intervention group

	**Mean**	**SD**	**Min**	**Median**	**Max**
**Previous training**					
Felling sluggish (pts. 1–6)	2.8	0.9	1.2	2.9	5.4
Psychological stress (pts. 1–6)	2.6	0.9	1.2	2.8	5.1
**After training**					
Felling sluggish (pts. 1–6)	2.2	0.9	1.1	2.0	5.0
Psychological stress (pts. 1–6)	2.1	0.8	1.1	1.9	4.6
Teatment effect (previous vs. after)	p < .001 in both parameters				
Difference T0-T2	p < .001 in both parameters				
Pain during intervention (n, %)		5 (16.7)			
Pain medication needed (n, %)		4 (13.3)			

This table shows the results of a questionnaire previous and after intervention respective feeling sluggish and psychological stress (pt 1 = least to pt 6 = most). Treatment effect (previous vs. after training) and difference in both parameters were significant (p < .001). Pain during intervention was documented according VAS-scale (0–10). Concomitant pain medication during intervention was evaluated (number of patients, %).

**Table 4:** Results of “chair stand test” and pain response

**Chair stand test**		**Intervention group (n = 30)**						**Control group (n = 30)**				
	**n**	**Mean**	**SD**	**Min**	**Median**	**Max**	**n**	**Mean**	**SD**	**Min**	**Median**	**Max**
Baseline (t0)	30	5.1	1.4	2.0	5.0	7.0	30	4.6	2.0	0.0	4.0	9.0
End of RT (t1)	30	6.7	1.9	4.0	6.5	12.0	30	4.9	2.2	0.0	5.0	10.0
After 3 month (t2)	25	9.0	2.6	5.0	10.0	13.0	23	5.0	2.7	0.0	5.0	10.0
Treatment effect within groups (t2)		p < .001						p = 0.525				
Treatment effect between groups (t2)		p < .001										
**pain response (VAS 0–10)**												
Pain score at baseline (VAS)	30	4.8	2.1	0.5	5.0	9.0	30	5.1	2.7	0.0	5.3	9.0
Pain score end of RT (VAS)	30	2.4	2.0	0.0	2.0	8.0	30	3.3	2.5	0.0	3.3	9.0
Pain score after 3 month (VAS)	25	1.9	1.4	0.0	1.5	5.0	23	3.8	2.3	0.0	4.5	7.0
Treatment effect within groups (t2)		p < .001						p = 0.010				
Treatment effect between groups (t2)		p = 0.003										
No response (n, %)	8	32%					12	52.2%				
Partial response (n, %)	5	20%					6	26.1%				
Complete response (n, %)	12	48%					5	21.7%				
Treatment effect (t2)		p = 0.158										

The “chair stand test” showed an improvement in intervention group over the course (p < .001), and between groups in favor to intervention group (p < .001). The intervention group improved in pain score (VAS, 0–10) over the course (p < .001), and was significant better between groups (p = .003) after 3 month. The overall pain response showed no significant difference between groups (p = .158). Wilcoxon U test was used between groups, signed-rank test was used within groups. Chi-square test was measured for pain response.

**Table 5:** Results of activity questionnaire of both groups

	**Intervention group**						**p-value**	**Control group (n = 30)**						**p-value**
	**Baseline (t0) (n = 30)**			**After 3 month (t2) (n = 25)**				**Baseline (t0) (n = 30)**			**After 3 month (t2) (n = 23)**			
**Q**	**Mean**	**Min**	**Max**	**Mean**	**Min**	**Max**		**Mean**	**Min**	**Max**	**Mean**	**Min**	**Max**	
1	2.5	1.0	6.0	1.8	1.0	5.0	0.063	3.6	1.0	6.0	3.2	1.0	6.0	0.022
2	2.6	1.0	6.0	2.0	1.0	6.0	0.137	3.4	1.0	6.0	3.6	1.0	6.0	0.028
3	3.5	1.0	6.0	3.6	1.0	6.0	0.490	3.8	1.0	6.0	3.1	1.0	6.0	0.255
4	2.3	1.0	6.0	1.9	1.0	6.0	0.011	3.6	1.0	6.0	3.1	1.0	6.0	0.063
5	2326.7	100	5000	2768.0	100	5000	0.061	1430.0	100	5000	1882.6	100	5000	0.109
6	3.0	1.0	6.0	2.8	1.0	6.0	1.000	2.9	1.0	6.0	2.6	1.0	6.0	0.791
7	0.4	0.0	1.0	0.5	0.0	1.0	0.426	0.3	0.0	1.0	0.4	0.0	1.0	0.548
8	2466.7	0.0	10000	4400.0	0.0	15000	0.708	3000.0	0.0	20000	3347.8	0.0	20000	0.478
9	1.3	0.0	6.0	1.6	0.0	6.0	0.848	1.6	0.0	6.0	1.8	0.0	6.0	0.945
10	2.8	1.0	6.0	2.6	1.0	6.0	0.008	4.1	1.0	6.0	3.7	1.0	6.0	0.080
11	3.8	1.0	6.0	3.3	1.0	6.0	0.675	4.0	1.0	6.0	3.3	1.0	6.0	0.974

The local control of metastases under treatment was 100% in both groups. In Arm A, no progression of other metastases in the vertebral column was seen after three months, while progression was recorded in 17.4% of the patients in Arm B (n = 4).

The intervention group improved in pain score (VAS, 0–10) over the course (p < .001), and was significant better between groups (p = .003) after 3 month. The results for complete pain response and partial response were 48% and 20%, respectively, in Arm A and 21.7% and 26.1% in Arm B. The overall pain response showed no significant difference between groups (p = .158) (Table 4).

**Figure 3:**
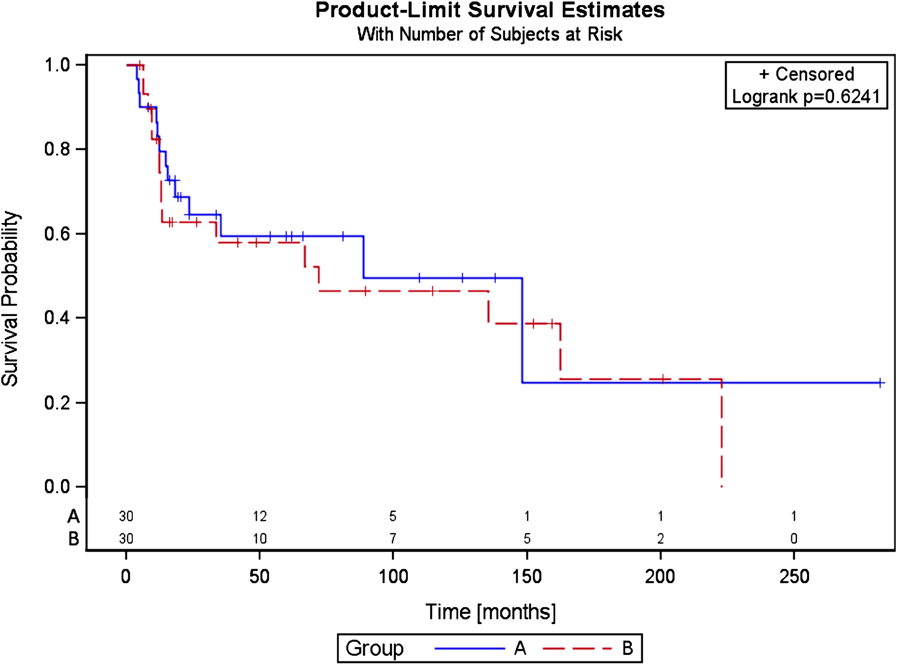
Overall survival of both arms, time in month.

The median overall survival of the intervention group was 88.6 months, six-month survival 90%, and twelve-month survival 83.1%. The median overall survival of the control group was 72 months, and six- and twelve-month survival 96.6% and 78.6%, respectively (p = .626). No statistically significant difference was observed (Figure 3). Median bone survival was 23.3 months in Arm A (range 2.1-52.0) and 11.2 months in Arm B (range 1.3-96.4) (p = .558).

## Discussion

Bone metastases are a very frequent secondary diagnosis associated with an advanced tumor disease, with the vertebral column being the most frequent localization [17,18]. Patients affected by this condition are usually immobilized, primarily due to the risk of pathological fractures and the related danger of spinal cord compression. Previous clinical studies have shown that tumor patients may profit from physical training measures during and following medical treatment [6,7,9,19,20]. Patients in the intervention group felt less exhausted and less psychically stressed following the training session; moreover, the pain felt during training was less intense. The specific enhancing effects of physical exercise, however, vary according to the degree of the primary disease, the medical treatment principles, and the patient’s current lifestyle [5]. The German Association for Sports Medicine and Prevention and the German Cancer Society have published guidelines for the design of training and sports programs for tumor patients; in these guidelines, the targeted sports intervention is deemed contraindicated in patients with bone metastases [21]. The promoting effects of differentiated training to support the vertebral column of patients with bone metastases have not yet been investigated. Current strength-training regimens with strong anabolic effects on muscles and bones may exert an influence in countering specific side-effects of tumor therapy, helping patients to improve their physical function [8]. A training exertion between 20 and 30% of maximum power causes neither an increase nor a decrease in strength, and can be seen as corresponding with the daily load of induced muscle tensions [22]. When a patient is immobilized, the muscles are exerted only to a degree not exceeding 20%, resulting in their atrophy [22]. The training threshold, thus, lies at approximately 30-40% of the maximum muscle strength, above which training can have a positive effect [22]. This was the level of exercise at which our training program was carried out without extra weights, although it was not possible to measure the maximum strength in these patients. In the intervention group, this training effect resulted in an increase in mobility, chair-stand test and activity questionnaire were used. The quantitative measurement of mobility for these patients was difficult, due the increased risk of pathological fracture. Therefore other methodical devices were not acceptable. We were not able to assess the strengthening of the muscles quantitatively, but this test was almost related to mobility for palliative patients. In addition mobility was evaluated in our non-validated questionnaire. The results of the activity questionnaire further emphasized the benefits attained in the intervention group as opposed to the control group. Other existing validated questionnaires had no information with respect to daily activity in patients with bone metastases, so we created questions independently which were relevant for these palliative patients. The questionnaire was not based on an existing one. However, this represented a major limitation. An adequate training duration corresponds to 20-30% of the time of muscle tension until exhaustion, and this was approximately the limit we used for the exercises [22]. Regarding age and gender, there are indications of differing degrees to which muscles can be trained; in our study group, however, due to the homogeneous distribution this difference appears to be negligibly small. Lasting only a few seconds, the individual muscle-tension is kept so short that no load is exerted on the cardiovascular system, meaning that these exercises can be carried out also by patients with pre-existing internal diseases. In their review, Knols et al. [5] demonstrated that the positive effects of exercise therapy vary depending on the type and stage of tumor, pharmaceutical therapy, therapeutical procedures, and patient lifestyle. In the review of the practicability it was not necessary to standardize the conditions, which is why simple-to-perform exercises were selected to form this standardized training program. On account of the raised risk of fracture, no extra weights were used and active movements of the vertebral column were avoided. As a measure to ensure an adequate training stimulus, which optimally lies at 40-50% of the maximum isometric strength with extra weights, the individual exercises were repeated a number of times, ensuring appropriate pauses between each set of exercises.

A decisive step was to initially classify the metastases as “stable” or “unstable”, which was done according to the Taneichi scores. According to Taneichi et al. [12], significant risk factors included the destruction of the costovertebral articulation, the size of the tumor in the thoracic region (Th1-Th10), and the destruction of the pedicle as the main factors in the thoracolumbar and lumbar spine. Not only is the standardized assessment of the stability in clinical practice by means of a score rating of relevance when making the indication for radiotherapy, it also provides important information for decisions regarding mobility therapy. The feasibility of exercise in tumor patients has already been demonstrated by a number of studies [9,10,19,20,23,24]. In their study Murnane et al. [25] were able to show that the majority of patients wish to take physical exercise as an adjunct to RT. In our investigation, none of the patients withdrew from training or refused to take part because of the training program. Hayes et al. [6] describe that physical exercise is associated with a benefit during and after tumor treatment, and indeed is even capable of reducing the impact of the side-effects of therapy and the symptoms of the underlying disease. The evidence emphasizes the effect of positive physiological and psychological benefits of mobility therapy during and after tumor therapy [8].

The local three-month controls were performed in 100% in both groups; the interesting long-term results have not yet been evaluated, and the results will be presented in the near future.

The pain-reducing effect in the three-month course of the study showed a positive course in Arm A, but not significantly better. In a prospective collective group of 518 patients, Chow et al. were able to demonstrate complete and partial response rates at the 3-month follow up of 21% to 25% and 26% to 30% in RT group, respectively [26]. Our results in the control group were comparable with these findings: the pain response in the intervention group was 48% and 20% in the three-month course.

There were no significant differences between the groups regarding overall survival (OS). Due to the differing tumor entities and the small number of patients involved, any comparison with other data cannot be representative. The bone survival data showed median values of 23.3 vs. 11.2 months; here, too, it was not possible to demonstrate a significant difference between the two groups.

The weak points of the study were the small number of subjects, the variety of primary tumors, the exclusion of the cervical spine, and the non-validated score of the activity questionnaire, purpose-made for this trial. The patients’ compliance with the training program in their home setting could naturally only be checked by reviewing the documentation forms completed by the patients themselves. The study’s strong points comprised the classification of stability and the very first application of a physical exercise program in patients with metastases in vertebral bodies as a measure to enhance their mobility.

## Conclusions

In this group of patients we were able to show that guided isometric training of the paravertebral muscles can be safely practiced in palliative patients with stable bone metastases of the vertebral column, improving their pain score and mobility. Large controlled trials are necessary to confirm these findings.

## Competing interests

The authors declare that they have no competing interests.

## Authors’ contributions

HR and JD developed and planned this trial. TB is responsible for statistical considerations/basis of the analysis. GO, MA, and TW estimated the stability of bone metastases. HR, SR, MH, DH, and IS performed the examinations and RT supervisions. HR and AG made the data collection. HR and FN performed the physical exercise. All authors read and approved the final manuscript.

## Pre-publication history

The pre-publication history for this paper can be accessed here:

http://www.biomedcentral.com/1471-2407/14/67/prepub
